# Glucose Sensing Neurons in the Ventromedial Hypothalamus

**DOI:** 10.3390/s101009002

**Published:** 2010-10-08

**Authors:** Vanessa H. Routh

**Affiliations:** Department of Pharmacology & Physiology, New Jersey Medical School (UMDNJ), Newark, NJ 07101, USA; E-Mail: routhvh@umdnj.edu; Tel.: +973-972-1489

**Keywords:** glucose-excited neurons, glucose-inhibited neurons, insulin, leptin, obesity, diabetes, hypoglycemia, hypoglycemia-associated autonomic failure, fasting

## Abstract

Neurons whose activity is regulated by glucose are found in a number of brain regions. Glucose-excited (GE) neurons increase while glucose-inhibited (GI) neurons decrease their action potential frequency as interstitial brain glucose levels increase. We hypothesize that these neurons evolved to sense and respond to severe energy deficit (e.g., fasting) that threatens the brains glucose supply. During modern times, they are also important for the restoration of blood glucose levels following insulin-induced hypoglycemia. Our data suggest that impaired glucose sensing by hypothalamic glucose sensing neurons may contribute to the syndrome known as hypoglycemia-associated autonomic failure in which the mechanisms which restore euglycemia following hypoglycemia become impaired. On the other hand, increased responses of glucose sensing neurons to glucose deficit may play a role in the development of Type 2 Diabetes Mellitus and obesity. This review will discuss the mechanisms by which glucose sensing neurons sense changes in interstitial glucose and explore the roles of these specialized glucose sensors in glucose and energy homeostasis.

## 1. Introduction

It is clear that the brain regulates energy homeostasis. The brain responds to circulating signals of nutrient status and adjusts food intake accordingly [1]. The brain also receives information regarding peripheral adiposity stores. In response to this feedback, the brain modulates the metabolism and/or insulin sensitivity of a variety of tissues including liver, skeletal muscle, and adipose tissue via the autonomic nervous system [2]. Thus, under normal conditions the brain senses peripheral energy status and responds by regulating the balance between energy intake, expenditure and storage in order to maintain body weight within a fairly tight range.

The autonomic regulation of energy homesostasis involves a coordinated effort between a number of interconnected brain regions including, but not limited to, the hypothalamus, amygdala and brainstem. In addition to brain regions associated with autonomic regulation, neurocircuitry related to reward pathways (e.g., nucleus accumbens) is also important for regulating food intake and body weight [1,3]. Thus, the neural systems involved in maintaining a stable body weight are extremely complex and much work remains to truly understand how these disparate brain regions interact to control energy homeostasis. The hypothalamus, in particular, has been the focus of numerous studies in the past century [4]. Within the hypothalamus, the ventromedial (VMH), dorsomedial (DMH), lateral (LH) and paraventricular (PVH) regions have all demonstrated importance [5]. The ventromedial hypothalamus (VMH) which consists of the ventromedial and arcuate nuclei (VMN and ARC, respectively) is one key region for integrating peripheral signals of nutrient status and adiposity. The ARC contains the neuropeptide Y (NPY) and pro-opiomelanocortin (POMC) neuronal populations which have opposing effects on energy homeostasis. NPY increases food intake and activates energy sparing mechanisms while melanocortins decrease food intake and increase energy expenditure [6]. The VMN also plays a role in regulating energy homeostasis however the exact neuronal populations involved are as yet unknown [4].

In addition to its response to circulating peptides and hormones which reflect energy status, the brain also senses and responds to changes in blood glucose levels. In the 1950’s, Jean Mayer’s glucostatic hypothesis [7] postulated that glucose receptors exist in the hypothalamus and possibly other central and peripheral regions known to be involved in the regulation of food intake. This hypothesis further stated that plasma glucose levels are sensed by these glucose receptors in the brain and that an increase in glucose utilization triggers meal initiation. The concept of hypothalamic glucose sensors has withstood the test of time. In fact, glucose sensors were first discovered in the VMH and LH [8,9]. The question as to whether these glucose sensors control daily food intake remains controversial. In 1986 Campfield and Smith showed that a decrease followed by an increase in blood glucose levels was correlated with meal initiation [10]. Moreover, interstitial glucose levels in the VMH and LH vary with blood glucose concentration [11]. These data suggest that brain glucose sensors could regulate daily food intake. However, Levin and colleagues recently showed that there was no correlation between VMH glucose levels and spontaneous feeding [12]. Thus, it is unlikely that VMH glucose sensors regulate meal to meal food intake although it does not rule out a role for glucose sensors in the LH or other brain regions. Interestingly, Levin and colleagues did find that falls in VMH glucose concentration in response to insulin-induced hypoglycemia were correlated with increased food intake. This observation is consistent with the important role of the VMH in the counterregulatory response that restores euglycemia following insulin-induced hypoglycemia [13–15]. Thus, VMH glucose sensors may play a role in detecting and countering severe glucose deficit. It stands to reason that the brain would respond to severe glucose deficit since glucose is the primary fuel of the brain.

Glucose sensors are located in many key areas of the brain (e.g., hypothalamus [9,16–19], nucleus solitarius [NTS] [20], amygdala [21]) which monitor energy status in the body and initiate appropriate sympathoadrenal and neurohumoral responses to maintain glucose and energy homeostasis. Glucose sensors are also located in peripheral tissues including the portal and mesenteric veins [22], carotid body [23] and intestine [24]. Glucose sensing neurons are defined as those which alter their action potential frequency in response to changes in interstitial glucose levels [18,25]. The effects of glucose on an individual hypothalamic neuron can be either postsynaptic (e.g., direct) or via an upstream presynaptic neuron (e.g., indirect) [18]. There are 2 main categories of neurons whose action potential frequency is directly (e.g., postsynaptically) regulated by glucose; those which respond to decreases in interstitial glucose below 2.5 mM [18] and those which respond to increases above 5 mM [26]. Both of these categories of glucose sensing neurons can be further divided into 2 subtypes. Glucose-excited (GE) neurons increase while glucose-inhibited (GI) neurons decrease their action potential frequency in response to increases in interstitial glucose from 0.1 to 2.5 mM glucose [18]. Similarly, high GE (HGE) and high GI (HGI) neurons increase or decrease their action potential frequency, respectively, in response to increases in interstitial glucose from 5 to 20 mM [26]. GE neurons require glucose metabolism for excitation[25,27,28]; however GI neurons comprise distinct populations which either require glucose metabolism [27,28] or sense the glucose molecule directly [29]. Whether glucose sensing by HGE or HGI neurons is dependent on metabolism is unknown.

In this review we will focus on the metabolically sensitive VMH GE and GI neurons as prototype glucose sensors for two reasons. First, VMH GE and GI neurons have been more thoroughly characterized in comparison to either the HGE/HGI neurons or glucose sensing neurons in other brain regions. Second, the glucose sensitivity of VMH GE and GI neurons lies within the range of interstitial glucose concentrations found within the majority of the brain *in vivo* under physiological and pathophysiological conditions (*i.e*., 0.2 to 5 mM) [30–33]. The evidence that interstitial brain glucose levels are approximately 30% of those found in the blood is as follows. Using a glucose oxidase electrode implanted in the VMH Silver and Erecinska demonstrated that at 7.6 mM plasma glucose in a fed anesthetized rat, interstitial VMH glucose was only 2.5 mM. Decreasing plasma glucose to 2–3 mM or increasing to 15 mM, resulted in brain glucose levels of 0.16 mM and 4.5 mM, respectively [11,34]. Although these studies were performed in anesthetized animals, they are consistent with recent studies in conscious animals. Using the zero net flux method for microdialysis, DeVries *et al*. show that VMH glucose levels were approx 1.5 mM in fed unanesthetized rats and 0.7 mM after an overnight fast [35]. Interestingly, using similar techniques, McNay and colleagues have shown that brain glucose levels vary with rat strain, brain region, and neuronal activity [36–39]. However, all of these studies consistently indicate that physiological levels of glucose within the brain vary within a fairly tight range from 0.7 to 2.5 mM. In fact, even though the ARC lies near the median eminence where the blood brain barrier is “leaky”, Dunn-Meynell *et al*. found that ARC interstitial glucose levels are indistinguishable from the rest of the VMH [12]. On the other hand, interstitial brain glucose levels below 0.7 mM and above 2.5 mM are associated with pathological hypo- and hyperglycemia, respectively [11]. Both VMH GE and GI neurons are extremely sensitive to glucose changes under 2 mM, with minimal response above 2 mM suggesting that these glucose sensing neurons primarily sense glucose deficit [30,31]. It is unclear whether brain glucose levels ever exceed 5 mM in the presence of an intact blood brain barrier. This raises questions about the physiological significance of HGE and HGI neurons. However, it should be noted that hyperglycemia impairs the integrity of the blood brain barrier [40]. Thus, these glucose sensing subtypes could contribute to hyperglycemia-associated pathology.

Therefore, the purpose of this review is to put forward the hypothesis that VMH glucose sensing neurons are part of a safety network which protects the brain against severe energy deficit. This hypothesis posits that glucose sensing neurons evolved as a defense against famine. In the modern world, they would also be important for restoring euglycemia following iatrogenic insulin-induced hypoglycemia. On the other hand, it would not be energetically efficient if small meal to meal changes in glucose levels altered the systems responsible for protecting the brain against the dangers of hypoglycemia. Thus, this hypothesis further posits that during normal energy homeostasis it would be important that these glucose sensing neurons do not respond to small glucose decreases associated with meal to meal fluctuations in blood glucose and activate the powerful homeostatic systems present in the VMH. This hypothesis leads to the prediction that obesity and type 2 diabetes mellitus (T2DM) are associated with a hypersensitivity of glucose sensing neurons to small glucose reductions. If glucose sensing neurons became overly sensitive to decreased glucose it could lead to an inappropriate signal in the brain of energy deficit. This would lead to activation of energy sparing mechanisms during conditions of energy sufficiency (or excess) in order to compensate for the perceived deficit. Such compensation could contribute to the development or exacerbation of obesity and T2DM. In support of this hypothesis, Colombani *et al*. recently showed that injection of a low glucose concentration into the carotid artery toward the brain caused a greater increase in VMH neuronal activity in obese fa/fa Zucker rats compared to their lean counterparts. This carotid glucose injection did not change peripheral glucose levels and thus reflects a central increase in sensitivity to glucose deficit in a rodent model of T2DM [41].

The following review is designed to evaluate the literature supporting such a role for VMH glucose sensing neurons. In order to accomplish this goal, it will first describe VMH glucose sensing neurons and the mechanisms by which they sense glucose. The role of VMH glucose sensing neurons during energy deficit (*i.e*., fasting, insulin-induced hypoglycemia) will then be discussed. Next, changes in VMH glucose sensing neurons during T2DM will be described. Finally, the data will be summarized and interpreted with regard to the proposed hypotheses.

## 2. Glucose Sensing Neurons

### 2.1. GE Neurons

GE neurons have been identified in the ARC and VMN [9,18,25,30,31,42]. Like the pancreatic β-cell, VMH GE neurons utilize the ATP-sensitive potassium (K_ATP_) channel to sense glucose. This was first demonstrated by Ashford and colleagues using single channel and whole cell patch-clamp recording techniques [17,25,43]. Many investigators using a variety of experimental approaches including molecular biology, pharmacology and transgenic animal models have corroborated these findings [18,28,30,44,45]. The concentration-response relationships for both K_ATP_ channel currents and action potential frequency of VMH GE neurons reveal a very steep and virtually linear relationship for glucose concentration and neuronal activity between 0.1 and 1.5 mM glucose. The slope of the curve then decreases sharply and plateaus between 2.5 and 5 mM glucose [30]. These data strongly suggest that the K_ATP_ channel on GE neurons plays a role in physiological brain glucose sensing. Moreover, they support a role for VMH GE neurons in the detection of energy deficit.

Pancreatic β-cells and GE neurons share several other components of glucose sensing. For example, the glucose transporter 2 (GLUT2), as well as glucokinase (GK), the rate limiting enzyme for glycolysis, are expressed in both GE neurons and β-cells [27,28,46]. Like the β-cell, GK plays a clear role in glucose sensing in GE neurons [27,28,46]. However, while K_ATP_ channels are expressed in all GE neurons, only approximately half of VMH GE neurons express GK and approximately 30% express GLUT2 [28]. Approximately half of VMH GE neurons also express the insulin sensitive glucose transporter, GLUT4 [28]. Claret *et al*. have shown that transgenic mice lacking the α2 subunit of AMP-activated protein kinase (AMPK), an important cellular fuel gauge, also lack ARC GE neurons [47]. However, we found that acute pharmacological activation or inhibition of AMPK had no effect on glucose sensing in VMH GE neurons [48]. These data suggest that multiple subtypes of GE neurons may exist which utilize alternate glucose sensing strategies.

The peptide phenotype of ARC and VMN GE neurons remains controversial. As mentioned previously, the ARC contains several important cell populations which play a role in the regulation of energy balance. ARC POMC and NPY neurons play a reciprocal role in energy homeostasis. Activation of POMC neurocircuitry favors decreased food intake and increased energy expenditure. In contrast, NPY leads to increased food intake and decreased energy expenditure [1]. Several studies support the hypothesis that GE neurons belong to the ARC POMC population. Electrical activity of POMC neurons is correlated with changes in energy status [49]. Both Ibrahim *et al*. and Claret *et al*. have demonstrated that POMC neurons were GE neurons using a transgenic mouse in which the POMC promoter was labelled with green fluorescent protein (GFP) although the glucose concentrations used by Ibrahim *et al*. were supra-physiologic [47,50]. On the other hand, several observations indicate that POMC neurons are not GE neurons. First, GE neurons are not present in the region of the ARC considered the location of the POMC neurons in either rats or wild-type mice. Rather, VMH GE neurons are concentrated in the cell-poor region located between the ARC and the VMN along the ventrolateral border of the VMN (VL-VMN) [30,48]. Second, immunohistochemical evaluation following electrophysiological recording in brain slices from rats has shown that GE neurons which respond to changes in interstitial glucose below 2.5 mM are not POMC neurons [30]. Moreover, POMC neurons from a different strain of POMC-GFP mouse than that used above were not glucose sensing [51]. Finally, glucose injection into the carotid artery did not increase levels of the marker of neuronal activation, cfos, in POMC neurons [51]. Whether these discrepancies represent differences between rat and mouse or between strains of transgenic mice remains to be determined before one can clearly conclude that POMC neurons are GE neurons. However, VMH GE neurons are involved in the NPY-POMC signalling network since NPY inhibits while melanocortin stimulating hormone (αMSH) stimulates GE neurons [30].

Although the phenotype of GE neurons remains elusive, the observation that these neurons are concentrated in the VL-VMN supports a role for these neurons in metabolic regulation. In the 1940s it was found that specific lesions which include the VL-VMN and the capsule surrounding the VMN cause the most profound obesity [52]. Later studies further support a critical role for this region in the regulation of energy balance. The neurotoxin gold-thioglucose which causes obesity and T2DM destroys neurons specifically in the VL-VMN and the “cell-poor” region between the VMN and ARC [53]. Moreover, many VMH efferent projections to other brain regions involved in metabolic regulation arise from the VL-VMN. A full discourse on the VL-VMN is beyond the scope of this review. However, an excellent and comprehensive review by Bruce M. King carefully evaluates the role of the VL-VMN in the regulation of energy balance in composite data dating to 1940 [4]. Because specific lesions of the VL-VMN cause obesity this area mostly likely supports increased energy expenditure and decreased energy storage. Unfortunately, due to a lack of anatomical markers and a full understanding of neuronal circuitry, the VL-VMN has been sorely neglected in recent years. However, clearly GE neurons are concentrated in a region of the VMH which has a well established role in the regulation of energy homeostasis.

In addition to being located in a region which is critical for energy homeostasis, VMH GE neurons are regulated by the peripheral adiposity signals, insulin and leptin. Leptin is a hormone secreted from white adipose tissue in proportion to the amount of adipose stores. Insulin levels, in addition to being regulated by blood glucose, also rise with adiposity. Both leptin and insulin play important roles in the endocrine feedback loop to the hypothalamus which signals the brain that peripheral energy stores are sufficient. Moreover, POMC neurons are activated and NPY neurons are inhibited by leptin and insulin. Thus, increased leptin and insulin levels decrease food intake and body weight [2]. Spanswick *et al*. found that insulin had a profound inhibitory effect on the activity of GE neurons due to K_ATP_ channel activation [42]. In contrast, while insulin still opened the K_ATP_ channel in 2.5 mM glucose, this change in conductance was insufficient to alter action potential frequency [48]. The lack of effect of insulin on action potential frequency in 2.5 mM glucose does not mean that insulin does not exert a physiological effect on GE neurons. Rather it suggests that under physiological conditions insulin regulates the responsiveness of GE neurons to presynaptic input or changes in the interstitial milieu. Insulin activation of K_ATP_ channels would increase whole cell conductance making it less likely that other inputs (or changes in glucose) would alter GE neuron’s membrane potential. Our recent data showing that insulin prevents GE neurons from detecting decreased interstitial glucose via the phosphoinositol-3-kinase (PI3K) signaling pathway support this concept [48]. Furthermore, these data are consistent with our hypothesis that under conditions of energy sufficiency, when insulin is present, the ability of GE neurons to sense decreased glucose would be masked. On the other hand, when insulin levels are low (e.g., after a fast) or when insulin resistance is present, GE neurons may respond to glucose decreases.

Leptin also regulates the activity of some GE neurons. Similar to their observations with insulin, Spanswick *et al*. showed that leptin in 10 mM glucose caused complete inhibition of VMH GE neurons by opening K_ATP_ channels [54]. However, in 2.5 mM glucose VMH GE neurons have a heterogeneous response to leptin, with some showing no response and others being inhibited [30] or excited [55]. These data suggest that not all GE neurons have leptin receptors. Indeed, while leptin receptors are abundant in the ARC and dorsomedial VMN, they are sparse in the lateral VMN [56]. Such a conclusion is again consistent with multiple subtypes of GE neurons. The effect of leptin on the glucose sensitivity of GE neurons has not yet been evaluated. However, both insulin and leptin open K_ATP_ channels on hypothalamic neurons via the PI3K signaling pathway [57]. Thus, we might predict that leptin also attenuates the response of certain populations of GE neurons (at least those which possess leptin receptors) to decreased glucose.

### 2.2. GI Neurons

Some of the components of glucose sensing in GI neurons are similar to those found in GE neurons. For example, Levin and colleagues have demonstrated a clear role for GK in VMH GI neurons [27,28,46]. Similarly, both GLUT2 and GLUT4 are expressed in a portion of VMH GI neurons [28]. However, the signal transduction pathway by which changes in intracellular ATP alter the activity of VMH GI neurons is completely distinct.

**Figure 1.**
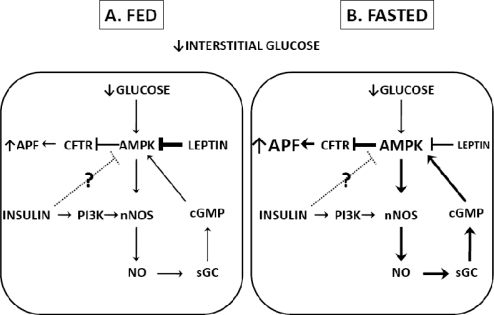
Regulation of the glucose sensitivity of NPY-GI neurons in fed and fasted conditions. Decreased interstitial glucose levels are translated into decreased intracellular glucose levels resulting in an increased AMP/ATP ratio. Increased AMP/ATP activates AMPK which enhances NO-sGC-cGMP signaling. Increased levels of cGMP are needed for full AMPK activation and closure of the CFTR chloride conductance in response to decreased glucose. CFTR closure leads to depolarization and increased action potential frequency. In the fed state, elevated leptin levels cause tonic inhibition of AMPK and attenuation of the response of GI neurons to decreased glucose. Insulin levels are also elevated in the fed state. Insulin increases nNOS activation *via* PI3K which could lead to enhanced responses to decreased glucose. It is also possible that insulin may inhibit AMPK in GI neurons since this effect of insulin has been demonstrated in hypothalamic tissue. AMPK inhibition would contribute to a low sensitivity to glucose decreases. However, the effects of insulin on the glucose sensitivity of GI neurons have not yet been evaluated. In the fasted state, both leptin and insulin levels are reduced. The reduction in leptin-induced AMPK inhibition leads to an enhanced response of GI neurons to decreased glucose. Whether insulin contributes to or opposes this change in glucose sensitivity is not known. Abbreviations: NPY (neuropeptide Y), GI (glucose-inhibited), AMPK (AMP-activated protein kinase), sGC (soluble guanylyl cyclase), cGMP (cyclic GMP), CFTR (cystic fibrosis transmembrane regulator), PI3K (phosphatidylinositol-3-kinase).

We have recently described the signaling pathway by which decreased glucose activates VMH GI neurons in detail [58]. This signaling pathway is shown in Figure 1A. The first step involves activation of the α2 subunit of AMPK. AMPK is a heterotrimeric protein which is activated by an increase in the AMP:ATP ratio, as well as by several upstream kinases including the tumor suppressor, LKB-1, and Ca^2+^/calmodulin dependent protein kinase (CaMKK), [59,60]. AMPK activation turns on catabolic processes which generate ATP while turning off anabolic ATP consuming processes. A role for AMPK in energy balance is well established (for review see [60]). Hypothalamic AMPK is a target for a number of hormones and transmitters which regulate energy balance [61–65]. Pharmacological activation of hypothalamic AMPK increases food intake [66]. Thus, it is not surprising that decreased glucose activates AMPK in GI neurons.

The next step in glucose sensing by GI neurons involves an interaction between AMPK and the gaseous messenger, nitric oxide (NO) [58]. VMH AMPK activation phosphorylates neuronal NO synthase (nNOS) leading to NO production in GI neurons [67]. NO, in turn, further activates AMPK via the NO receptor soluble guanylyl cyclase (sGC) which increases cyclic GMP (cGMP) levels. Amplification of AMPK activation by the sGC-cGMP pathway is necessary for depolarization of GI neurons in response to decreased glucose [58]. Such an interaction has also been described in skeletal muscle [68] and vascular endothelial cells [69]. In endothelial cells cGMP activates the upstream AMPK kinase, CaMKK. CaMKK further activates AMPK for a full biological effect [69]. These data lead to the hypothesis that, as in endothelial cells, the NO signaling pathway in GI neurons activates an upstream AMPK kinase such as CAMKK. AMPK kinase activation increases the sensitivity of AMPK for AMP [65]. Thus, the NO signaling pathway may be a critical determinant of the glucose sensitivity of GI neurons.

As mentioned above role for AMPK in energy balance is well established [64,65,70–72]. Although less lauded, NO strongly deserves consideration. NO is a unique neurotransmitter due to its ability to diffuse across cell membranes [73]. Furthermore, nNOS is a potential site whereby many neurotransmitters and hormones could influence the glucose sensitivity of GI neurons. nNOS activity is dependent on, and enhanced by intracellular calcium increases which are spatially localized to this enzyme [74]. Thus, any neurotransmitter which is linked to a calcium permeable ion channel or increases calcium release from internal stores in such a way to spatially engage nNOS could potentially enhance the response of GI neurons to decreased glucose. nNOS is also a target of a number of hormones, including leptin and insulin [67]. Moreover, a number of observations suggest that NO regulates energy balance. Orexin-, ghrelin- and NPY-induced feeding are NO dependent [75–77]. NOS activity and expression are elevated in obese *ob/ob* mice which lack the leptin receptor, and NOS inhibition decreases food intake and body weight in these animals [78]. This latter observation supports our hypothesis that under normal energy status, it is important that glucose sensing neurons do not respond to small changes in interstitial glucose. However, during diabetes these neurons may become sensitized to glucose decreases. NO-mediated increases in the sensitivity of AMPK for AMP would enhance the response of GI neurons to glucose decreases. Thus, NOS inhibition would be expected to normalize food intake and body weight in *ob/ob* mice. The *in vivo* data of Colombani *et al*. showing increased neuronal activation in response to low glucose in fa/fa Zucker rats, which have a dysfunctional leptin receptor, are consistent with the hypothesis that GI neurons have an enhanced response to glucose decreases in T2DM [41].

The last step in the response of VMH GI neurons to decreased glucose involves closure of a chloride channel [18,51]. We hypothesize that the identity of this chloride channel is the cystic fibrosis transmembrane regulator (CFTR) [51,58]. AMPK phosphorylates and inhibits the CFTR [79]. Gemfibrozil, which selectively binds the CFTR, blocks the effects of low glucose by opening a chloride channel [51,58]. Furthermore, decreased glucose, AMPK activation and the cell permeable cGMP analog, 8-bromo-cGMP all increase VMH CFTR phosphorylation, while nNOS inhibition blocks CFTR phosphorylation in response to decreased glucose [58]. However, gemfibrozil has been reported to have opposing effects on the peripheral CFTR [80]. Moreover, while AMPK phosphorylates and inhibits the CFTR [79], phosphorylation is not always correlated with channel closure [81]. Thus, further studies are needed to confirm a role for the CFTR as the specific channel mediating glucose sensing by VMH GI neurons. Interestingly, there is a high incidence of diabetes among patients with cystic fibrosis; however it is not clear whether this is due entirely to peripheral CFTR dysfunction or whether there is a central component [82–85].

Like GE neurons, leptin and insulin regulate VMN GI neurons. Insulin increases NO production by VMH GI neurons via the PI3K signaling pathway [67]. In contrast, leptin inhibits NO production in VMN GI neurons in 2.5 mM glucose by inhibiting AMPK [33]. Leptin also attenuates the response of GI neurons to decreased glucose. The AMPK activator, 5-aminoimidazole-4-carboxamide-1-b-4-ribofuranoside (AICAR), blocked leptin’s effect on the glucose sensitivity of GI neurons. Moreover, leptin prevents the increase in VMH AMPKα2 phosphorylation in response to decreased glucose [33]. This is consistent with reports by others showing that leptin inhibits hypothalamic AMPK activity [64]. The effect of insulin on glucose sensitivity of GI neurons has not been evaluated. Since insulin increases NO production in GI neurons, it is possible that insulin enhances their response to decreased glucose. On the other hand, insulin hyperpolarizes GI neurons possibly by activating KATP channels [31,67]. This latter effect may attenuate activation in response to decreased glucose. To further complicate the matter, insulin has also been shown to inhibit hypothalamic AMPK [64]. Thus, it is difficult to predict insulin’s effect on glucose sensitivity. However, our observation that leptin blocks the response to decreased glucose is again consistent with our overall hypothesis that under conditions of energy sufficiency (e.g., in the presence of leptin) the ability of GI neurons to sense glucose decreases is blocked.

## 3. Glucose Sensing Neurons and Energy Deficit

### 3.1. Fasting

It is well established that the ARC NPY neurons which project to the PVN [86] as well as the spinal cord [5] stimulate food intake and decrease energy expenditure. Approximately 40% of NPY neurons are GI neurons [33,51,87]. Moreover, fasting enhances both the response of VMH GI neurons and NPY release to glucose decreases [33]. Thus, the stimulatory effect of fasting on NPY release is mediated, in part, by changing the glucose sensitivity of NPY-GI neurons such that they are activated by smaller glucose decreases. AMPK activation shifts the glucose sensitivity of GI neurons from fasted animals to that seen in GI neurons from fed animals while AMPK inhibition does the converse [33]. Leptin inhibits hypothalamic AMPK and the response of VMH GI neurons to decreased glucose [33,64]. Thus, decreased leptin levels during fasting may contribute to the enhanced response of NPY-GI neurons to decreased glucose by releasing the normal AMPK inhibition [1,64]. Figure 1 illustrates the hormonal regulation of GI neurons in the fed and fasted state. These data suggest that NPY-GI neurons release more NPY in response to glucose deficit when peripheral energy stores (e.g., glycogen) are depleted. Thus, in the fasted state where brain energy levels are diminished and leptin levels are low, glucose deficit activates NPY-GI neurons to a greater extent. Increased activation of NPY-GI neurons may lead to increased food intake and decreased energy expenditure in order to restore energy balance. However, if this system were inappropriately activated during energy sufficiency, it could also contribute to the development of T2DM and obesity.

### 3.2. Hypoglycemia Detection and the Counterregulatory Response (CRR)

Intensive insulin therapy prevents the deleterious effects of hyperglycemia in Type 1 (T1) and advanced T2DM. The major side effect of intensive insulin therapy is hypoglycemia. Powerful neuroendocrine and autonomic counterregulatory responses (CRR) that prevent and correct hypoglycemic conditions protect the brain from hypoglycemia [88,89]. The CRR involves the release of glucagon, epinephrine and corticosterone. Together these hormones restore euglycemia. However, these mechanisms can become impaired as a result of recurrent hypoglycemic episodes, leading to a life-threatening condition known as hypoglycemia-associated autonomic failure (HAAF). During HAAF, the glycemic threshold for the CRR shifts to lower glucose levels and the mechanisms that provide warning signals of impending hypoglycemia (increased sweating, confusion, *etc*.) become severely impaired. As a result, glucose levels are allowed to drop, without detection, to dangerously low or lethal levels [88]. There are significantly more episodes of severe hypoglycemia in T1DM. However, hypoglycemic episodes are on the rise in T2DM and the risk of severe hypoglycemia increases with duration of the disease [90–92]. Thus, recurrent hypoglycemia is currently a major limiting factor in the management of T1 and T2DM.

Glucose sensors in the portal vein, the brainstem and the VMH have all been linked to counterregulatory responses to hypoglycemia and/or glucoprivation [14,93–95]. The relative roles of these regions in the CRR are still not completely understood. The rate of glucose decline may determine whether peripheral or central sensors predominate. When glucose levels decrease rapidly, central sensors appear to play the dominate role whereas the response to a slower decline is mediated by glucose sensors in the portal and mesenteric veins [93]. In humans with T1DM, glucose declines occur at variable rates during a 24 hour period. Hypoglycemia develops more slowly overnight or in response to basal insulin replacement but can be rapid after meals especially with exercise and/or rapidly acting insulin analogues (R. McCrimmon, University of Dundee, Scotland UK, personal communication). This suggests that both central and peripheral glucose sensors play a role in the response to hypoglycemia. Moreover, it is likely that the initiation of the CRR involves integration between multiple brain and peripheral sensors [96]. The range of glucose concentrations to which VMH GI and GE neurons respond suggest that they sense glucose deficit [18,30]. Thus, glucose sensing neurons may play a role in protecting the brain against dangerous glucose decreases.

Observations showing that the ability of VMH GI and GE neurons to sense decreased glucose is impaired under a number of conditions in which the ability of the brain to mount the CRR is also impaired support a role for these neurons in hypoglycemia detection and the CRR. First, as mentioned above, recurrent hypoglycemia impairs the CRR and produces HAAF. The ability of VMH GI and GE neurons to sense glucose decreases is also attenuated by recurrent hypoglycemic episodes [32,97,98]. Second, VMH Injection of the corticotrophin-releasing factor 2 receptor agonist, urocortin, impairs the CRR *in vivo* and urocortin incubation *in vitro* attenuates the response of VMH GI and GE neurons to decreased glucose [95]. Third, VMH lactate infusion also impairs the CRR [99]. When glucose is lowered in the presence of lactate, VMH GI neurons have a blunted response to decreased glucose (GE neurons have not yet been evaluated) [32]. Finally, VMH NO signaling is necessary for both the full generation of the CRR and glucose sensing by VMH GI neurons. VMH NOS inhibition *in vivo* and nNOS inhibition *in vitro* impair the CRR and the response of GI neurons to decreased glucose, respectively [58,100]. Interestingly, the CRR is impaired and VMH GI neurons are completely absent in mice lacking nNOS [100]. Similarly, T1DM is associated with an impaired CRR and VMH GI neurons from T1DM rats neither produce NO nor depolarize in response to decreased glucose [36,101]. Together, these data provide strong evidence that VMH GI and GE neurons play a role in hypoglycemia detection. Moreover, these data indicate that alterations in the glucose sensitivity of VMN glucose sensing neurons may contribute to HAAF.

**Figure 2.**
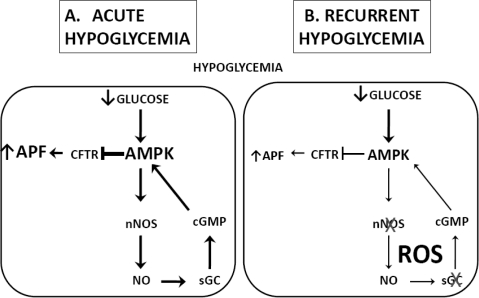
Hypothetical mechanism underlying effects of recurrent hypoglycemia on the glucose sensitivity of VMH GI neurons. Acute hypoglycemia (A) leads to activation of AMPK and the NO-sGC-cGMP signaling pathway. cGMP further activates AMPK leading to closure of the CFTR, depolarization and increased action potential frequency. Hypoglycemia also increases ROS levels. The combination of NO and ROS production may cause S-nitrosylation of nNOS and sGC. S-nitrosylation of these enzymes leads to a decrease in their activity and a reduction in NO signaling. Therefore, recurrent hypoglycemia (B) would lead to decreased sensitivity of GI neurons to reduced glucose. Abbreviations: VMH (ventromedial hypothalamus), GI (glucose-inhibited), AMPK (AMP-activated protein kinase), sGC (soluble guanylyl cyclase), cGMP (cyclic GMP), CFTR (cystic fibrosis transmembrane regulator), ROS (reactive oxygen species).

The mechanism by which hypoglycemia impairs VMH GI and GE neurons glucose sensitivity is not known. One possibility is that hypoglycemia increases nutrient flux in neurons and masks glucose deficit [102–106]. Alternatively, hypoglycemia may impair glucose responsive signaling by glucose sensing neurons. One mechanism by which this may occur in GI neurons is the production of reactive oxygen species (ROS). For example, insulin-induced hypoglycemia increases brain ROS levels [107]. When NO is produced in the presence of ROS there is an increase in S-nitrosylation of a number of proteins including sGC and nNOS [108]. S-nitrosylation of these enymes decreases their activity [109,110]. As discussed above, impaired NO signaling prevents GI neurons from sensing glucose deficit. This hypothesis which is illustrated in Figure 2 is consistent with the observation that RH decreases nNOS activity [98]. Our preliminary data showing that preventing ROS production during insulin-induced hypoglycemia prevents the subsequent development of HAAF provide further support for this hypothesis [111]. Clearly much more work needs to be done to understand how hypoglycemia impairs central glucose sensing. Understanding the mechanisms by which VMN GSNs detect hypoglycemia and their dysfunction after recurrent hypoglycemia will facilitate development of appropriate treatments for diabetes aimed at maintaining tight glycemic control while reducing the incidence of inadvertent hypoglycemia.

An interesting and unanswered question relates to the specific aspects of the CRR affected by GI and/or GE neurons. GI neurons are a heterogeneous population in that 40% of ARC NPY neurons are GI neurons [33,51,87]. However VMN GI neurons are obviously not NPY neurons since this phenotype does not exist in the VMN [112]. Studies of NPY KO mice showed that feeding in response to insulin-hypoglycemia was absent however hormonal counterregulation was unaltered [113]. This suggests that the NPY-GI neurons may belong to the population of NPY neurons which project to the PVH and play a role in food intake [112]. nNOS-KO mice that lack GI neurons in the mediobasal hypothalamus (ARC + VMN) showed significantly decreased epinephrine (but not glucagon) responses to hypoglycemia suggesting that VMN GI neurons are involved in the sympathoadrenal cCRR [100]. It is possible that the glucagon response was not significantly impaired in these animals due to the presence of normal GE neurons. We have shown that GE, as well as GI, neurons are impaired under conditions where both epinephrine and glucagon responses to hypoglycemia are impaired [32,98]. Taken together, these data suggest that different populations of glucose sensing neurons may play distinct roles in the CRR. However, it is clearly premature at this point to go beyond casual speculation.

## 4. Glucose Sensing Neurons and T2DM

**Figure 3.**
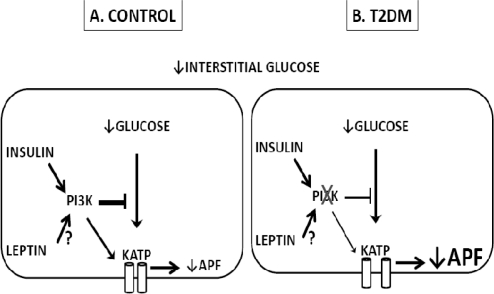
Hormonal Regulation of the glucose sensitivity of VMH GE Neurons. Hormonal regulation of the glucose sensitivity of VMH GE neurons in controls (A) and T2DM (B). Decreased interstitial glucose levels are translated into decreased intracellular glucose. This decreases the ATP/ADP ratio, opens the KATP channel and hyperpolarizes the cell leading to a decrease in action potential frequency. Insulin, via PI3K, attenuated KATP activation in response to decreased glucose. Since PI3K also mediates leptins effects on GE neurons, it is possible that leptin similarly reduces GE responses to decreased glucose however this has not been tested. Moreover, leptin is known to have pleiotropic effects on VMH GE neurons. In T2DM, insulin resistance leads to an increased responsiveness of GE neurons to glucose decreases which is normalized by enhancing PI3K signaling. Whether leptin resistance during T2DM contributes to enhanced responses of GE neurons to decreased glucose remains to be tested. Abbreviations: VMH (ventromedial hypothalamus), GE (glucose-excited), T2DM (type 2 diabetes mellitus), KATP (ATP-sensitive potassium channel), PI3K (phosphatidylinositol-3-kinase).

T2DM is associated with central insulin and leptin resistance [114,115]. Insulin and leptin prevent VMH GE and GI neurons, respectively, from sensing glucose deficit [33,48]. Thus, it is logical to hypothesize that VMH glucose sensing neurons might be inappropriately responsive to glucose decreases during T2DM. This is now known to be true for VMH GE neurons [116]. GE neurons from diabetic Zucker fa/fa rats are insensitive to the effects of insulin on neuronal activity [42]. Similarly, VMH GE neurons from diabetic db/db mice respond to glucose decreases in the presence of insulin, while VMH GE neurons from wild-type mice do not. Moreover, the baseline response of VMH GE neurons from db/db mice to glucose decreases is enhanced compared to VMH GE neurons from wild-type control mice. Both baseline glucose sensitivity as well as the effect of insulin on VMH GE neurons from db/db mice is restored by the insulin sensitizer, Compound 2. The effects of Compound 2 are blocked by PI3K inhibition [116]. This latter observation is consistent those indicating that T2DM is associated with dysfunctional hypothalamic PI3K signaling [2]. Thus these data support our hypothesis that GE neurons have an enhanced response to decreased glucose in T2DM. The regulation of the glucose sensitivity of VMH GE neurons by insulin is illustrated in Figure 3.

At this time it is not possible to determine with any certainty whether altered glucose sensing in GE neurons is a contributing factor in the development of T2DM or whether it results from T2DM. However, there is some evidence supporting a causal role for GE neurons in the development of T2DM and obesity. Rats that are predisposed to develop diet-induced obesity (DIO) have a pre-existing impairment in hypothalamic insulin and leptin sensitivity [117,118]. Moreover, reducing hypothalamic PI3K activity leads to reduced energy expenditure and weight gain [119]. These data suggest that deficits in hypothalamic insulin signaling, and thus impaired glucose sensing in GE neurons may precede the development of obesity and diabetes. One caveat that should be mentioned with regard to our hypothesis is that Levin *et al*. showed that the increase in fos-like immunoreactivity in response to glucose is reduced in DIO-prone rats compared to those rats which resist DIO [120]. However, this study specifically investigated increased glucose whereas the present hypothesis is related to responses to decreased glucose. Moreover, GE neurons themselves show very little response above 2.5 mM glucose, so it is possible that the glucose-induced neuronal activation in response to elevated glucose in these rats was due to a different population of glucose sensing neurons.

The role of GI neurons during T2DM is difficult to predict. Colombani *et al*. recently showed that low glucose injected toward the brain increased ARC electrical activity to a greater extent in fa/fa Zucker rats compared to their lean counterparts [41]. This is consistent with enhanced responses of GI neurons to decreased glucose. Furthermore, leptin clearly attenuates the response of GI neurons to decreased glucose [33]. Thus, leptin resistance in DIO-prone or Zucker fa/fa rats could cause enhanced responses of VMH GI neurons to decreased glucose. On the other hand, the CRR and the response of GI neurons to decreased glucose were attenuated in the neuronal insulin receptor knockout (NIRKO) mouse [121]. This discrepancy may reflect the relative contribution of leptin vs. insulin signaling in these neurons. As mentioned above, leptin inhibits the response of GI neurons to decreased glucose by inhibiting AMPK [33]. While insulin has also been reported to inhibit hypothalamic AMPK [64], its PI3K-mediated effects on NO production appear to dominate in GI neurons [67]. Thus, insulin might be predicted to increase the response to glucose deficit as a result of its stimulatory effect on NO production in these neurons [67]. Our data showing that GI neurons are more sensitive to glucose deficit in the fasted state when both leptin and insulin levels are low suggest that leptin’s effect on AMPK outweighs that of insulin on NO production [33]. However, it remains to be seen whether the effect of leptin or insulin resistance will play the dominant role in the function of VMH GI neurons in T2DM. The only thing that is clear is that, like GE neurons, GI neurons are regulated by both leptin and insulin and therefore one would predict that their function would be altered during the hypothalamic leptin and insulin resistance which precedes the development of DIO.

## 5. Summary

In summary, the data provided above support the overall hypothesis that the metabolically sensitive VMH glucose sensing neurons serve to protect the brain during times of severe glucose deficit. VMH GI and GE neurons are exquisitely sensitive to changes in glucose below euglycemia with little response above. VMH NPY-GI neurons become more sensitive to decreased glucose in the fasted state. This change in the glucose sensitivity of NPY-GI neurons is correlated with increased NPY release in response to decreased glucose. Thus, these data suggest that glucose sensing neurons probably evolved to protect the brain during times of famine. Glucose sensing neurons also appear to be important for detecting hypoglycemia and generating the CRR. Thus, enhanced detection of glucose deficit during famine would also protect against the dangers of hypoglycemia. Moreover, the impaired CRR following antecedent hypoglycemia may result, in part, from impairments in the ability of glucose sensing neurons to sense glucose decreases. VMH GI and GE neurons clearly have the potential to regulate metabolic homeostatic neurocircuitry. In the ARC, 40% of NPY neurons are GI neurons. GE neurons are concentrated in the VL-VMN, a region long associated with obesity and T2DM. The observation that fasting enhances NPY release in response to decreased glucose suggests that the existence of NPY-GI neurons is physiologically significant. Increased NPY neurotransmission during fasting would increase food intake and decrease energy expenditure to compensate for energy deficit. However, it is important that such powerful homeostatic mechanisms are not called into play by small decreases in blood glucose associated with meal to meal changes. Thus, it is logical that leptin and insulin prevent GI and GE neurons, respectively, from sensing glucose decreases. Finally, a prediction derived from this hypothesis is that glucose sensing neurons are hypersensitive to glucose deficit during T2DM. Such enhanced sensitivity would lead the brain to conclude that energy deficit existed in the presence of energy sufficiency or even excess. An inappropriate signal of energy deficit could lead to compensatory signals contributing to the development or exacerbation of obesity and T2DM. The data showing that VL-VMN GE neurons are indeed hypersensitive to glucose decreases in T2DM supports this interpretation. Moreover, increased neuronal activity in response to low glucose *in* vivo in T2DM suggests that VMH GI neurons may also be more sensitive to glucose decreases during diabetes and obesity [41]. Therefore, taken together, these data suggest that VMH glucose sensing neurons sense glucose deficit. When these neurons “turn a blind eye” to glucose deficit, impairments in the CRR result. Similarly, one might predict that disease related anorexia-cachexia might also be associated with diminished sensing of glucose decreases. On the other hand, when glucose sensing neurons “cry wolf” (e.g., are too sensitive to glucose deficit) the brain misinterprets energy sufficiency or even excess and overcompensates.

While this hypothesis is compelling and well supported by the data presented, a number of questions remain. First and foremost, the data linking VMH GI and GE neurons to hypoglycemia detection and initiation of the CRR are circumstantial. The direct connections between glucose sensing neurons and the autonomic nervous system are not known. It is true that some NPY neurons are GI neurons. However, the GI neurons in the VMN are clearly not NPY neurons. It is clear that determining the peptide and neurotransmitter phenotype of both VMN GI and VL-VMN GE neurons is critical. Though, it is likely that glucose sensing neurons will form heterogenous populations belonging to a number of metabolic “emergency” circuits to be called into service when needed. It will also be important to determine how glucose sensing neurons become impaired during HAAF and how that might be rectified. Even more important is the need to understand how the glucose sensitivity of VMH glucose sensing neurons is maintained within a range that allows protection during energy deficit without causing overcompensation during energy sufficiency. Resolving these issues will likely lead to improved treatments for T1 and T2DM/obesity.

Finally, it is important to note that this hypothesis pertains only to the metabolically sensitive VMH GE and GI neurons. A role for other glucose sensing neurons in daily food intake and energy homeostasis remains to be established. What is clear is that VMH GE and GI neurons are not responsive to changes in glucose which would occur between meals, especially in the presence of insulin and leptin [30–33,48]. However, glucose concentration-response relationships have not been determined for other subtypes of glucose sensing neurons. Moreover, it is well established that glucose injections towards the brain *in vivo* increase hypothalamic neuronal activation (*i.e*., fos-like immunoreactivity) [51,120]. Similarly, increased brain glucose leads to insulin secretion [122] and inhibits feeding [123]. Thus, the brain is able to detect and respond to increases in glucose above euglycemia. This response could be mediated by either neuronal or astrocytic glucose sensors which are regulated by glucose metabolism or by sensing the glucose molecule directly[51,122–125].
